# Age and mental health moderate the association between environmental concern (EC) and smoking frequency: smoking as a polluting behavior

**DOI:** 10.3389/fpubh.2023.1089148

**Published:** 2023-10-04

**Authors:** Weixi Kang

**Affiliations:** Department of Brain Sciences, Imperial College London, London, United Kingdom

**Keywords:** age, mental health, environmental concern, moderation, smoking frequency

## Abstract

It is well-recognized that smoking is detrimental to the environment. However, much less is understood about smoking behavior from an environmental perspective with a focus on environmental concern (EC). This study aims to establish the association between EC and smoking frequency in smokers and test whether age and mental health moderate such an association. Obtained by analyzing data using regressions on smokers (*N* = 3,599) from Understanding Society: the UK Household Longitudinal Study (UKHLS), which is a representative sample in the UK, the results revealed that age and mental health moderate the association between EC and smoking frequency. This association is important to understand because smoking pollutes the environment, and very few studies have looked at smoking behavior from an environmental perspective.

## 1. Introduction

A total of 6 trillion cigarettes are produced every year (1). Among these 6 trillion cigarettes, 5.8 trillion cigarettes are consumed by smokers worldwide (1, 2). Although smoking prevalence has decreased in developed nations (3), global consumption of cigarettes continues to grow as a result of smoking in young people in developing countries (4). Most smokers will agree that smoking not only poses a serious health risk (5) but also is detrimental to the environment. Although important, surprisingly, few studies have looked at environmental attitudes in relation to smoking.

### 1.1. The detrimental effect of smoking on the environment

Smoking brings not only negative health consequences but also environmental issues, which include “the use of scarce arable land and water for tobacco cultivation, use of harmful chemicals on tobacco farms, deforestation, and carbon emissions from manufacture and distribution processes to the production of toxic waste and nonbiodegradable litter”. Moreover, incorrect disposal of cigarette butts is associated with numerous domestic and wildland fires with devastating consequences (2).

From an individual level, according to a recent study (2), “a typical smoked cigarette stick was shown to have a water footprint of 3.7 L, a fossil fuel use equivalent to 3.5 g of oil, and a climate change impact of 14 g of CO^2^ equiv emissions. Over a lifetime, a person smoking a pack a day for 50 years has a carbon footprint of 5.1 t CO^2^ equiv, which would require 132 tree seedlings grown for 10 years to offset. Their water footprint of 1,355 m^3^ is equivalent to almost 62 years' supply for any three people's basic hygiene and food hygiene needs, and the lifetime fossil fuel depletion of 1.3-ton oil equiv is comparable to the electricity use of an average household in India for almost 15 years”. Additionally, cigarette smoking can be several times greater than the resource depletion and pollution caused by other types of commodity consumption if comparing the footprint of such a smoker (7.3 kg of tobacco consumption per year) to the global average sugar (24.3 kg) and red meat (14.4 kg) consumption per capita per year. Specifically, a smoker contributes 5 times more to water depletion, which is about 2 and 10 times more to the fossil fuel depletion caused by consuming sugar and red meat, respectively, and also contributes 4 times more to climate change than a sugar consumer. Together, these pieces of evidence suggest that smoking is detrimental to the environment.

### 1.2. Environmental concern (EC)

Previous studies have operationalized EC in several non-mutually exclusive ways (6). For instance, EC can reflect more fundamental factors such as religious beliefs and post-materialist principles. Moreover, some EC concepts refer to the fact that people are not concerned about the effect of environmental changes on themselves but are worried about the effects of environmental degradation on the health and wellbeing of people and other species around the world. Finally, EC can be understood as concerns about the severity of various environmental issues, the impact of human activities on these issues, and the support for solving these issues (7). The current research focuses on the third construct of EC, which is the most accepted definition of EC.

### 1.3. Barriers between EC and pro-environmental behavior

There are a lot of factors that hinder people from acting environmentally friendly even though they might have a high level of EC [e.g., (8–12)]. For instance, previous studies have found some situational factors such as the lack of access to green products (13) and some cultural factors (14) that make it hard for people to behave according to their pro-environmental attitude.

However, even without these situational constraints, people can still fail to act according to their environmental attitude (10). Another well-established explanation regarding why people with high pro-environmental attitudes engage in pro-environmental behavior is that the opportunity cost of performing environmental behavior is low (15–18). This notion helps to explain the reason why pro-environmental attitudes can better predict “low-cost” pro-environmental behavior (19) but often fail to explain “high-cost” behaviors such as driving or flying less (16, 20).

On the other hand, it has been theorized that people are more likely to engage in pro-environmental behavior when the perceived environmental benefits associated with the cost are high (21). For instance, in a scenario where people must choose to travel by bicycle or by car, they would wonder if it is worth the cost of a comfortable car ride to gain benefits for the environment (22). Thus, if the potential environmental benefit is low, then people would probably travel by car. Indeed, there is a lot of empirical support for this theory (22–25).

### 1.4. Age and EC

Age can serve as a moderator in the association between EC and smoking frequency for several reasons. Younger adults tend to have more EC than older adults (26, 27), or they may just experience qualitatively different EC compared to older generations [as suggested in a review by Fritze et al. (28)]. Moreover, with an increase in age, regard for the future tends to decrease (29). Thus, thoughts and care about the future are weakened compared to young people. Indeed, if EC is a type of concern regarding negative consequences in the future, then people who think about the future will reasonably have much more EC than older adults. Moreover, as young individuals live longer and may be alive to see the negative outcome and environmental change, it is expected that younger individuals may have greater EC. As such, younger people with higher EC may be more motivated to adopt healthier behaviors, including a decreased likelihood of smoking. In contrast, older individuals may have been exposed to different social norms and attitudes toward smoking, leading to a weaker association between their EC and smoking frequency. Second, age-related developmental factors play a role. Younger individuals are often more open to new experiences, including adopting pro-environmental attitudes and behaviors. In contrast, older individuals may have established long-standing smoking habits that are resistant to change, irrespective of their level of EC.

Consequently, interventions targeting older adults may need to employ strategies that acknowledge and address potential resistance or skepticism toward environmental issues. Tailoring interventions to the specific age-related differences in EC and incorporating intergenerational dialogue and education can help bridge the gap and enhance the effectiveness of smoking reduction interventions within different age cohorts.

H1: Age moderates the associations between EC and smoking frequency, and EC is negatively related to smoking frequency in younger smokers, whereas older smokers' smoking frequency is less or not affected by EC.

### 1.5. Mental health and EC

Mental health was defined by the World Health Organization (WHO) as a condition of wellbeing in which everyone fulfills their potential, can cope with the usual demands of life, can work successfully and fruitfully, and can contribute to their community (30). Mental health is closely related to smoking, and the intention of smoking for a lot of smokers is to reduce mental health-related problems. Indeed, several studies have found that people with various mental health issues are more likely to be smokers, heavy smokers, and heavily reliant on cigarettes (31–41).

Mental health can moderate the association between EC and smoking frequency through various mechanisms. Individuals with poor mental health may resort to smoking as a coping mechanism to deal with stress and negative emotions (42), regardless of their level of EC. Furthermore, mental health problems are related to worse emotional regulation (43), making it challenging for individuals to translate their EC into constructive actions. Motivation and self-efficacy, key factors in behavior change, can be undermined by mental health issues [e.g., (44)], hindering individuals from quitting smoking or adopting pro-environmental behaviors despite their EC. Additionally, compromised mental health can reduce an individual's level of awareness and engagement with environmental issues, potentially weakening the association between EC and smoking frequency.

H2: Mental health moderates the associations between EC and smoking frequency, and EC is negatively related to smoking frequency in people with better mental health, whereas the frequency of smoking in people with worse mental health remains unaffected by EC.

### 1.6. The current study

Thus, smoking is a form of non-environmental behavior as opposed to pro-environmental behavior. In addition, smoking is a unique case of non-environmental behavior characterized by its addictive mechanisms. It remains unclear if EC affects the frequency of smoking and whether age and EC moderate such relationships. The current study aims to test the above-stated hypotheses in the specific context of the United Kingdom, given that cultural differences and socioeconomic factors can significantly influence the relationship between EC and smoking frequency in smokers. Cultural norms and values shape individuals' attitudes and behaviors toward smoking, with some cultures exhibiting more acceptance or tolerance toward smoking than others. Moreover, socioeconomic factors such as income, education, and occupation can play a crucial role in shaping smoking habits and access to resources for smoking cessation. For instance, individuals from lower socioeconomic backgrounds may face higher barriers to quitting smoking due to limited access to healthcare services and cessation support. Additionally, cultural factors may interact with socioeconomic status, leading to variations in the importance placed on EC and their impact on smoking behavior.

## 2. Methods

### 2.1. Data

Data were extracted from Understanding Society: the UK Household Longitudinal Study (UKHLS), which has been collecting annual information from the original sample of UK households since 1991 [it was previously known as The British Household Panel Study, BHPS; (45)]. This dataset is publicly available at www.understandingsociety.ac.uk. All data collections were approved by the University of Essex Ethics Committee. Participants completed informed consent before participating in the study. The current study used data in Wave 10, which were collected between 2018 and 2019. Only smokers who have indicated their smoking frequency and do not have any missing variable of interest were left for further analysis. Thus, 3,599 smokers with a mean age of 44.61 ± 16.46 years ranging from 16 to 90 years of age were left for further analysis. In addition, on average, they smoked 11.42 ± 9.16 cigarettes per day.

### 2.2. Measures

#### 2.2.1. Smoking frequency

Smoking frequency was measured by the question to smokers: “Approximately how many cigarettes a day do you usually smoke, including those you roll yourself?” Participants answered this question with numbers. Thus, smoking frequency was standardized (mean = 0, SD = 0.97).

#### 2.2.2. EC

Participants were asked to indicate their concerns toward the environment with a Likert scale ranging from 1 (“Strongly agree”) to 5 (“Strongly disagree”) based on four statements: (1) “The so-called ‘environmental crisis' facing humanity has been greatly exaggerated”. (2) “If things continue on their current course, we will soon experience a major environmental disaster”. (3) “The effects of climate change are too far in the future to really worry me.” (4) “Climate change is beyond control–it's too late to do anything about it.” The internal consistency of these items as indicated by Cohen's alpha is 0.7. A higher factor score means more EC.

#### 2.2.3. GHQ-12

The GHQ-12 is a widely used instrument to measure mental health (46). As indicated by its name, there are 12 items in GHQ-12. The bi-modal scoring approach was used (47). In the bi-modal scoring system, each of the 12 items in the GHQ-12 is scored as follows: 0 points: If the response to an item is “Better than usual” or “Same as usual”. 1 point: If the response to an item is “Less than usual” or “Much less than usual”. Scores are reverse-coded when appropriate. After scoring each item, the total score is calculated by adding up the individual scores for all 12 items. Thus, the highest possible score is 12, whereas the lowest possible score is 0. A higher GHQ-12 score means worse mental health.

#### 2.2.4. Demographic variables

Demographic variables including age, sex, monthly income, highest educational qualification, and marital status were included in the analysis. Specifically, age and monthly income were coded as what they were (continuous), sex was coded as male (1) vs. female (2), highest educational qualification was coded as below college (1) vs. college (2), marital status was coded as single (1) vs. married (2), and residence was coded as urban (1) and rural (2).

### 2.3. Analysis

#### 2.3.1. Factor model

A confirmatory factor analysis (CFA) with one pre-specified factor was performed on MATLAB 2018a the MATLAB “factoran” function based on the four questions asked about EC with a customized script. This factor score was kept for further analysis. A higher score in EC means that participants are more concerned about the environment compared to a lower score. The factor loadings for questions (1) to (4) are 0.66, −0.41, 0.79, and 0.55, respectively.

#### 2.3.2. Hierarchical regression model

A hierarchical regression model was used to analyze the data and test the moderation effect of age and sex (48). Specifically, EC score after the factor analysis, the GHQ-12, age, and other demographic variables including sex, monthly income, highest educational qualification, marital status, and residence were entered in the model as predictors in the first step, and then age ^*^ EC and GHQ-12 ^*^ EC interactions were added to the model in the second step. Smoking frequency was the predicted variable in the model.

## 3. Results

**Figure 1:**
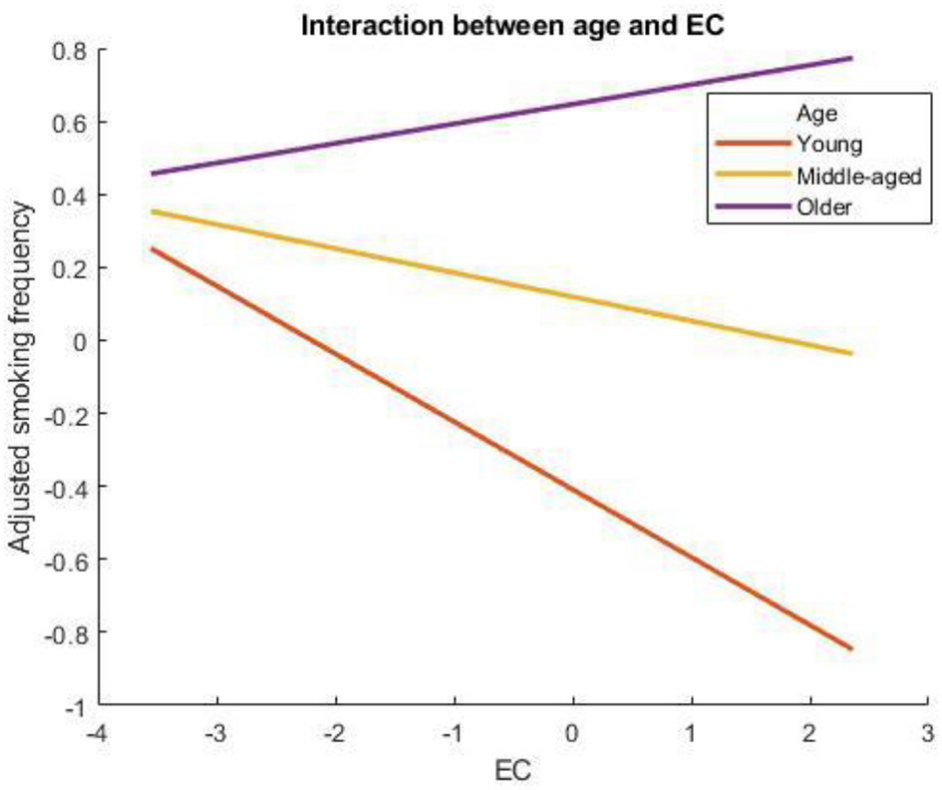
Age moderates the association between EC and smoking frequency. A higher score on the x-axis indicates more EC, whereas a higher score on the y-axis indicates more smoking frequency.

The whole regression model with interactions explained 9.88% of the variance in smoking frequency (Table 1). The current study found that age and mental health moderate the association between EC and smoking frequency (*b* = 0.003, *p* < 0.001, 99% C.I. [0.001, 0.005]). As shown in Figure 1, the negative relationship between EC and smoking frequency was strong (*b* = −0.19, *p* < 0.001, 95% C.I. [−0.25, −0.14]) for younger adults (−1 SD), less strong but significant for mean age adults (*b* = −0.06, *p* < 0.01, 95% C.I. [−0.09, −0.02]), and was not significant for older adults (+1 SD).

**Table 1:** The hierarchical regression coefficient (*b*) for step 1 uses demographics, mental health, and EC to predict smoking frequency, and step 2 adds age by EC and mental health by EC interactions with the total explained variances (R^∧^2).

Step 1	Age	0.01 [0.01, 0.02]^***^
	Sex	−0.16 [−0.22, −0.10]^***^
	Monthly income	0.00 [0.00, 0.00]
	Highest educational qualification	−0.22 [−0.29, −0.14]^***^
	Marital status	0.00 [−0.02, 0.03]
	Residence	0.02 [−0.06, 0.09]
	GHQ-12	0.02 [0.01, 0.03]^***^
	EC	−0.09 [−0.12, −0.06]^***^
	R^∧^2	0.0942
**Step 2**	Age	0.01 [0.01, 0.02]^***^
	Sex	−0.16 [−0.22, −0.10]^***^
	Monthly income	0.00 [0.00, 0.00]
	Highest educational qualification	−0.21 [−0.29, −0.14]^***^
	Marital status	0.00 [−0.02, 0.03]
	Residence	0.02 [−0.06, 0.09]
	GHQ-12	0.02 [0.01, 0.03]^***^
	EC	−0.26 [−0.34, −0.18]^***^
	Age^*^EC	0.003 [0.002, 0.005]^***^
	GHQ-12^*^EC	0.007 [0.0001, 0.01]^*^
	R^∧^2	0.0988

All numbers were rounded up to two decimal places. ^*^p < 0.05, ^**^p < 0.01, ^***^p < 0.001.

**Figure 2:**
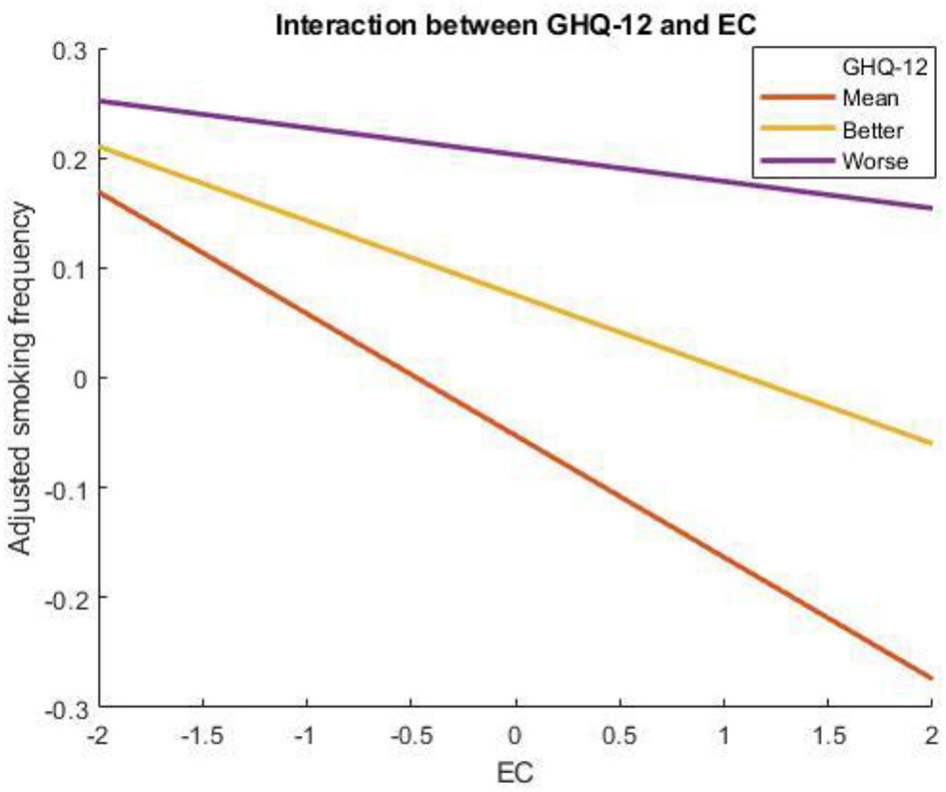
Mental health moderates the association between EC and smoking frequency. A higher score on the x-axis indicates more EC, whereas a higher score on the y-axis indicates more smoking frequency.

Mental health also moderated the negative association between EC and smoking frequency (*b* = 0.007, *p* < 0.001, 99% C.I. [−0.002, 0.017]; Figure 2). The negative association between EC and smoking frequency was strong (*b* = −0.08, *p* < 0.001, 95% C.I. [−0.12, −0.04]) for people with better mental health (−1 SD), a little bit stronger for people with mean level of mental health (*b* = −0.14, *p* < 0.001, 95% C.I. [−0.19, −0.09]), and not significant for people with worse mental health (+1 SD).

## 4. Discussion

The current study aimed to investigate the association between EC and smoking frequency, which is largely ignored in the literature given that smoking is a polluting behavior since studies have only focused on the health and addictive aspects of smoking, and no one has looked at smoking from an environmental perspective. The current research has contributed to the literature by demonstrating that EC is negatively related to smoking frequency, and this association is moderated by age and mental health, thus proposing that the factors of smoking behavior should include EC.

Smoking is detrimental to the environment [see Zafeiridou et al. (2) for a review] at both the macro (e.g., the use of scarce arable land and water for tobacco cultivation) and micro levels (e.g., CO2 emission by each cigarette one smokes). Thus, understanding the determinants of smoking behavior should not only come from sociodemographic factors, peer influence [e.g., (49)], pressure and stress [e.g., (50)], and mental health (51), but also should take environmental attitudes into account.

The finding that age moderates the negative association between EC and smoking frequency is largely consistent with the notion that younger adults have more EC than older adults (26, 27), given that they live longer and are more likely to experience the negative consequences brought by the environmental changes. Thus, older adults who had more EC did not influence the frequency of smoking, whereas EC was negatively associated with smoking in young and middle-aged people. Moreover, this association was stronger for young people compared to middle-aged people, which can also be explained by the fact that young people live longer than middle-aged people, and thus they want to protect the environment by having less smoking frequency when their EC is high. In addition, this result can also be potentially explained by the fact that young people are more likely to quit smoking successfully compared to old people because of young people's potentially lower levels of addiction and fewer years of living with tobacco as a regular part of their daily lives [e.g., (52–55)]. In the context of the current study, young people with high EC smoked less compared to older people with higher EC.

In addition, mental health as measured by GHQ-12 also moderates the negative association between EC and smoking frequency. Specifically, EC did not relate to the smoking frequency in smokers with worse mental health but was negatively related to people with better mental health. This finding can be explained by the fact that mental health is negatively related to smoking (31–41). Moreover, smokers with better mental health are more likely to quit [e.g., (56)]. Thus, smokers with fewer mental health problems smoke less when their EC is high to protect the environment.

While this research provided novel findings regarding how age and mental health moderate EC to predict smoking frequency in smokers, there are some limitations. First, the current sample did not include participants who were below the age of 16 years, and future research into the younger population should assess the potential moderating effect of age and mental health, including the possibility of a qualitatively different makeup of their EC (28). Specifically, adolescents are at the forefront of climate activism and expressed concerns about the future of the planet (57). A recent survey reported that four out of five young people aged between 14 and 23 years reported that they are “somewhat” or “very” anxious about climate change. Moreover, 17% of them reported that they lost sleep because they were worrying about climate change (58). One study also found that adolescents had high levels of concern about EC in an open-ended survey, with comments such as feeling “scared the earth will not last” [(59), p. 3]. Thus, future studies should consider the potential unique effect of EC on adolescents. Second, all the measures in the current study are self-reported, and future studies should use more objective measures such as biological assays for smoking frequency to see if the current conclusion still holds. Third, it is important to acknowledge that focusing on the third construct of EC may limit the scope of generalizability to that specific dimension. However, this study provides valuable insights into this particular aspect of EC and offers a foundation for future research to build upon. It is recommended that future studies incorporate multiple dimensions of EC to enhance the generalizability of findings across different contexts and populations. Fourth, the effect sizes were quite small in the current study, which should be borne in mind when interpreting the results of the current study. Finally, one possibility is that predictors in the models may serve as mediators in the association between EC and smoking frequency, which is not tested by the current research. Thus, it is important for future research to address this.

In sum, the results provided novel findings regarding how EC is related to smoking frequencies in smokers and how age and mental health moderate such an association. This association is important to understand because smoking pollutes the environment, and very few studies have looked at smoking behavior from an environmental perspective. This study may imply that increasing smokers' concerns may decrease their smoking frequency. Moreover, this intervention may be made while taking age and mental health into account as smokers in different age groups and with different levels of mental health may react to EC differently.

## Data availability statement

The original contributions presented in the study are included in the article/supplementary material, further inquiries can be directed to the corresponding author.

## Ethics statement

The studies involving humans were approved by University of Essex Ethics Committee. The studies were conducted in accordance with the local legislation and institutional requirements. Written informed consent for participation in this study was provided by the participants' legal guardians/next of kin.

## Author contributions

The author confirms being the sole contributor of this work and has approved it for publication. WK: conceptualization, data curation, formal analysis, investigation, methodology, resources, software, writing—original draft, and writing—review and editing.
